# Community Health Workers Improve HIV Disclosure Among HIV-Affected Sexual Partners in Rural Uganda: A Quasi-Experimental Study

**DOI:** 10.9745/GHSP-D-21-00631

**Published:** 2022-10-31

**Authors:** Zubair Lukyamuzi, Ruth Mirembe Nabisere, Rita Nakalega, Patience Atuhaire, Hajira Kataike, Bashir Ssuna, Mazen Baroudi, Flavia Matovu Kiweewa, Philippa Musoke, Lisa M. Butler

**Affiliations:** a Makerere University–Johns Hopkins University Research Collaboration, Kampala, Uganda.; b School of Public Health, College of Health Sciences, Makerere University, Kampala, Uganda.; c Infectious Diseases Institute, College of Health Sciences, Makerere University, Kampala, Uganda.; d Uganda Tuberculosis Implementation Research Consortium, Kampala, Uganda.; e Department of Clinical Epidemiology and Biostatistics, College of Health Sciences, Makerere University, Kampala, Uganda.; f Department of Epidemiology and Global Health, Umeå University, Umeå, Sweden.; g Institute for Collaboration on Health, Intervention, and Policy, University of Connecticut, Storrs, CT, USA.

## Abstract

**Background:**

We evaluated the efficacy of a community health worker (CHW)–led intervention in supporting disclosure among adults living with HIV in heterosexual relationships.

**Methods:**

We conducted a quasi-experimental study with 2 arms allocated by geographically determined clusters and adjusted for between-group differences among adults living with HIV in the greater Luwero region of Uganda who had never disclosed their status to their current primary sexual partners. Clusters were allocated to either a CHW-led intervention or a control arm. In both arms, participants were consecutively recruited. As opposed to receiving routine care for the control arm, participants in the intervention arm received additional CHW disclosure support. The overall follow-up was 6 months, and the primary outcome was disclosure to the sexual partner. Data were analyzed using a clustered modified Poisson regression model with robust standard errors to determine independent factors associated with disclosure.

**Results:**

Of the 245 participants who enrolled, 230 (93.9%) completed the study, and 112 (48.7%) of those were in the intervention arm. The median age was 30 (interquartile range=25–37) years, the majority were women (76.5%), and most (80%) did not know their partners’ HIV status at study entry. At the end of follow-up, the overall disclosure prevalence was 74.4% (95% confidence interval [CI]=68.2, 79.9) and participants in the intervention arm were 51% more likely to disclose compared to those in the control (adjusted relative ratio [aRR]=1.51; 95% CI=1.28, 1.77). Men were 24% (aRR=1.24; 95% CI=1.07, 1.44) more likely to disclose compared to women, and membership in an HIV/AIDS association increased disclosure by 18% (aRR=1.18; 95% CI=1.01, 1.39).

**Conclusion:**

CHW support improved disclosure among adults living with HIV in heterosexual relationships when compared to routine care. Therefore, CHW-led mechanisms may be utilized in increasing disclosure among adults living with HIV in heterosexual relationships in rural settings.

## Abstract (precis)

HIV disclosure is critical to achieve positive HIV treatment and management outcomes. Community health worker–led mechanisms may be used to support disclosure among adults living with HIV in heterosexual relationships in rural settings.

## Teaser

**Key Findings:**

- Community health workers used community-based counseling, home visits, phone calls, and disclosure skills building to support adults living with HIV in the disclosure process, which led to increased disclosure.
- Having community health workers able to support the disclosure process among adults living with HIV can help expand community-based HIV care and management for those experiencing difficulties with sexual partner disclosure or barriers to accessing health facilities or trained health care workers.

**Key Implications:**

- In addressing challenges of HIV treatment and management adherence in low-resource settings, policy makers should leverage community health worker programs to integrate HIV disclosure mechanisms for adults living with HIV in heterosexual relationships.
- In addressing HIV disclosure challenges among sexual partners, HIV care programs in low- and middle-income countries should advocate for community-based disclosure mechanisms located near populations with low attendance at health facilities.

## INTRODUCTION

HIV remains a major public health problem worldwide. Despite the world’s commitment to end HIV/AIDS by 2030,^1^^,^^2^ about 680,000 AIDS-related deaths and 1.5 million new infections occurred in 2020; this caused a persistent slow decline in new infections to fewer than 500,000, which was the 2020 global target.^3^^,^^4^ Despite contributing only 12% of the world’s population, sub-Saharan Africa bears the highest global HIV burden, with 71% of the world’s people living with HIV (PLHIV).^5^

HIV infection affects all age groups but is more prevalent among those who are sexually active or have ever been married.^5^ Among sexually involved couples in Uganda, 10% are affected by HIV, and of these, only 3% are concordant positive (both partners are HIV positive).^5^ In the management of HIV, disclosure is a critical challenge affecting both concordant and discordant couples.^6^^,^^7^ Apart from its prevention and care benefits,^8^^,^^9^ disclosure promotes social support, retention in care, and a sense of well-being by enhancing trust and social acceptance.^10^^,^^11^ Failure to disclose is associated with poor antiretroviral therapy (ART) adherence, development of treatment-resistant strains, and increased HIV transmission.^12^^–^^14^ Disclosure can be either planned or unplanned, and it should be understood as a process rather than a one-time event.^15^ Many PLHIV who are willing to disclose anticipate negative reactions such as blame, abandonment, violence, and separation. However, only a few such adverse events occur following disclosure.^15^^,^^16^ Disclosure can be done by the HIV-positive person themselves or by others, such as health workers on behalf of the HIV-positive person following their consent.^17^

Multiple factors influence disclosure in a sexual relationship.^18^^,^^19^ These include both barriers and the specific needs of the relationship, such as financial and social support.^17^^,^^20^^,^^21^ Other factors influencing disclosure include: literacy, number of sexual partners, index testing at antenatal care or voluntary counseling and testing centers, being on ART, receipt of disclosure counseling, time spent in HIV care, membership in an HIV/AIDS association, perceived level of stigma and discrimination, having responsibility to disclose, presence of a disclosure opportunity, and knowing the partner’s HIV status.^8^^,^^9^^,^^11^^,^^17^ Relatedly, disclosure varies greatly among sexual partners, but it is generally lower with casual partners than with steady partners. The duration taken to disclose also varies widely from the time of HIV diagnosis to after many years of living with the disease.^8^ Occasionally, disclosure is influenced by social desirability which is evidenced by the fact that 15.4% and 6.7% of men and women, respectively, claim to have disclosed when they actually have not.^22^ This form of deception in HIV care among PLHIV may lead to persistent undesirable HIV management outcomes.^22^

Despite the fundamental role of disclosure in improving HIV treatment and management outcomes,^23^^,^^24^ there are limited interventions to support disclosure among adults living with HIV in sexual relationships. The use of trained personnel who are qualified to offer professional health services in support of disclosure has been shown to be effective.^25^ However, it’s challenging to only rely on this approach, especially in low- and middle-income countries where there is a scarcity of trained professional health cadres. Additionally, men’s attendance at health facilities in sub-Saharan Africa is still very low despite vigorous efforts to encourage and remind women to always bring their spouses for HIV-related services.^26^ This has created a need for more readily available community-based interventions that are located geographically closer than health facilities to where people live.

Despite the fundamental role of disclosure in improving HIV treatment and management outcomes, there are limited interventions to support disclosure among adults living with HIV in sexual relationships.

In response to the scarcity of trained health professionals, particularly in low- and middle-income countries, the use of community health worker (CHW) programs reemerged as a desired strategy to increase access to health services.^27^^–^^29^ Uganda adopted the strategy in 2001 to bridge the health service delivery gap between the community and health facilities, and most villages now have 1 CHW serving at least 5–10 households.^30^ According to the country’s CHW operations guidelines, CHWs are selected by the community members themselves through a popular vote. They are trained on the job and undergo continuous refresher and update training organized by the Ministry of Health and partners. CHWs are entitled to a monthly stipend of 18000 Ugandan shillings (US$5), facilitation in terms of transport and lunch, and incentives such as certificates and T-shirts. They are responsible for conducting home visits, mobilizing communities for utilization of health services, promoting health in their communities, following up on people who have been discharged from health facilities and those on long-term treatment, and linking community members such as HIV-positive people to health services.^30^

CHWs’ effectiveness and contributions to improving access to health services and achieving desirable outcomes in HIV care have been documented.^31^^,^^32^ For example, CHW-based mechanisms have improved psychosocial outcomes in PLHIV, including self-efficacy, quality of life, social support, and reduced stigma.^33^ Yet despite CHWs being the most-approached health workers in the community by PLHIV,^32^^,^^34^ their specific role in supporting HIV status disclosure among adults living with HIV in sexual relationships is not clearly documented. Therefore, the main objective of this study was to evaluate the role of CHWs in supporting disclosure among adults living with HIV in heterosexual relationships in the greater Luwero district, a region with some of the highest rates of HIV prevalence and sexual partner nondisclosure in Uganda.^35^^–^^37^

## METHODS

### Study Design

**FIGURE 1:**
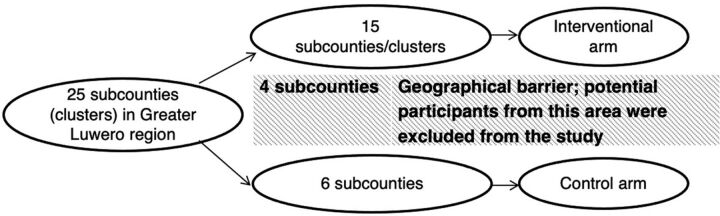
Recruitment of Adults Living With HIV From Their Respective Clusters in Rural Uganda

This was a quasi-experimental study with 2 study arms allocated by clusters. Clusters were subcounties of the greater Luwero region in Uganda that had been previously determined according to the geographical boundaries. From October 3, 2019 to May 31, 2020, the study was conducted among adults living with HIV in sexual relationships who had never disclosed to their sexual partners. Participants from geographically close clusters were allocated either an intervention (CHW support) or control arm (without CHW support), and the proportion of disclosure was compared between the study arms at the end of the 6-month follow-up. Because the intervention required interaction between participants and CHWs within the community, some clusters were utilized as a geographical barrier (buffer zone) between the intervention and control clusters, and all potential participants from the buffer zone were excluded from the study (Figure 1).

### Study Area and Population

The study area was the greater Luwero region, located about 20 kilometers from Kampala, the capital of Uganda, and includes 3 subdistricts: Luwero, Nakaseke, and Nakasongola districts. The region includes 25 subcounties, which served as clusters in this study. In Uganda, a subcounty is composed of about 6 parishes (each consisting of several villages or zones) with a subcounty chief in charge of technical matters (e.g., planning and mechanical or scientific work) and an elected local council III chairman. The region had a population of about 949,100,^35^ served by 3 government hospitals, 1 private not-for-profit hospital, and 7 health center IVs. In Uganda, a health center IV is managed by a doctor and provides minor surgical services and other general health services, and a hospital provides services for all surgeries and medical care.^38^^,^^39^ Therefore, the study sites were high-volume HIV care units in health facilities, namely Luwero, Nakaseke, and Kiwoko hospitals; Semuto, Nyimbwa, Kalagala, Ngoma, Nakasongola, Nabiswera, and St. Francis health center IVs. However, we were not able to work in the government military facility (one of the hospitals in the region) because it did not provide us with administrative approval until near the end of the project period. In addition to HIV care services, all study sites offered general health services.

Of the 25 clusters, 15 adjoining clusters were allocated to the intervention arm, 6 to the control, and 4 formed the buffer zone. Because of the prior administratively determined geographical sizes, clusters in the control arm were relatively bigger, and thus fewer than those in the intervention. Participants from the clusters were enrolled in the study arms in which their respective clusters had been allocated. Study participants were adults living with HIV who had been in heterosexual relationships for at least 3 months and had not disclosed their status to their current primary partner.

### Data Collection Procedures

For both study arms, participants were recruited between October 3, 2019 and November 7, 2019. All adults living with HIV who came in for HIV services at the study sites during the above period were informed about the study by the study focal person at the facility. Potential participants who were interested and readily available for the study were screened, and those who met the eligibility criteria were consented to and enrolled. The eligibility criteria were: adult (above 18 years), HIV positive irrespective of ART status, being in a heterosexual relationship for at least 3 months, having not disclosed their HIV status to their current primary sexual partner, having stayed in the study area (greater Luwero region) for at least 3 months, and willing to provide informed consent. Potential participants who were coming from the buffer zone were excluded from the study to minimize contamination. Participants were consecutively recruited by the study research assistants, who were HIV care providers (e.g., counselors) at the study sites. About 8 participants were recruited per day across all sites.

All participants completed a questionnaire at enrollment and a disclosure assessment form at 3-month and 6-month clinic visits. Participants in different study arms who were enrolled at the same study site were enrolled on different dates and given different appointment dates for subsequent visits to minimize cross-contamination at the study site. This was necessary because 2 sites (Luwero and Kiwoko hospitals) enrolled participants in both arms. Overall, Luwero hospital enrolled 48 participants (36 in the intervention arm and 12 in control) and Kiwoko hospital enrolled 33 participants (26 in the intervention arm and 7 in control). The rest of the other facilities enrolled 20 participants on average in either the intervention or control arm. All participants continued with routine HIV care, including HIV disclosure counseling, but those in the intervention arm received CHW support in addition.

The study end points were HIV disclosure or study end period (6 months), whichever came first. Those who experienced adverse events upon disclosure or during the study period were kept in the study up to the end of the follow-up, as they continued to receive reconciliation counseling (dispute resolution in case the partner was reachable), and social support from the study team and the health facility.

#### CHW Intervention

CHWs are members of their home communities and have basic training in providing basic health care services, including home visiting, health promotion and education, disease surveillance, mobilization for immunization services, follow-up with pregnant women and lactating mothers, and supporting HIV care services.^29^ For HIV services in particular, CHWs support linkage to care among those that need HIV services and provide basic HIV counseling, home-based care, health education, adherence support, and livelihood psychosocial support.^29^^,^^40^

CHWs support linkage to care among those that need HIV services and provide basic HIV counseling, home-based care, health education, adherence support, and livelihood psychosocial support.

After enrollment, each participant in the intervention arm was asked to provide the name and contact information (if they were known to the participant) of a CHW in their area of residence. The details of a CHW obtained from a participant were verified in the list of CHWs from the district registry. The 3 districts in the greater Luwero region were among the 112 districts with a CHW program, and the entire region had approximately 2,000 CHWs, which was consistent with the distribution of CHWs in the country.^29^

Verified CHWs were contacted and informed about the study and were scheduled for training. A total of 48 CHWs aged between 25 and 60 years were recruited and trained for 3 days. The training covered refresher HIV basic counseling skills; HIV status disclosure skills; health ethics; confidentiality and privacy; and management of adverse events associated with disclosure, such as domestic violence and separation. Trainings used both role play and didactic models, moderated by HIV care counselors and study investigators. Pre- and post-training assessments were completed.

In addition to the routine care, participants in the intervention arm were linked and attached to a trained CHW from their area of residence or its vicinity. One hundred and twenty-one participants were paired with 48 CHWs irrespective of gender in the ratio of 3:1. The participant and a CHW initially met and laid out a specific disclosure plan, which generally included 2 weekly phone calls and scheduled home visits. Discussions during phone calls and home visits included methods or skills to be used in disclosure, assessment of the partner’s attitude toward HIV and their personality, potential adverse outcomes and how to overcome them, and the partner’s availability and timings at home. They also practiced how to start and handle the disclosure process. Depending on the agreements from the discussions, eventual disclosure would occur at the participant’s home or at the health facility, according to the participant’s preference.

At the participant’s home, either the participant would disclose to their partner in the presence or absence of a CHW, or, in the presence of the participant, the CHW would disclose to the participant’s partner on the participant’s behalf. If done at the health facility, a CHW would encourage and arrange for couples’ HIV testing and counseling at the health facility of the participant’s choice. During couples’ testing and counseling at the facility, eventual disclosure would be done by the counselor on the participant’s behalf.

The CHW received ongoing supervision via regular phone calls and meetings with the study team. They completed home visit and phone call logs whenever they visited or telephoned the participant for study purposes, and they received a monthly facilitation and transport allowance of 50000 Ugandan shillings (US$15).

#### Control (Routine Care)

Participants continued to receive standard of care at their respective HIV care centers (i.e., the study sites), which involved HIV counseling and disclosure counseling at every routine care appointment visit, antiretroviral drug refills, adherence counseling, and psychosocial support. With routine care, participants would disclose at home by themselves or persuade their partners to go to the health facility for couples’ HIV counseling and testing, where eventual disclosure would occur with the help of a health worker.

#### Outcomes

The primary outcome was HIV disclosure at the end of the study follow-up. This was assessed at 3 monthly subsequent in-clinic study visits for every participant. Participants who disclosed were encouraged to bring their partners to the study site or health facility for further counseling and possible HIV testing. Secondary outcomes were occurrence of adverse events after disclosure (e.g., separation, fighting, and quarreling), lost to follow-up, and partner HIV testing following disclosure.

The primary outcome was HIV disclosure at the end of the study follow-up.

Independent variables included age, education, gender, marital status, nature of marriage, duration of the relationship, monthly income, partner HIV status, duration on ART, condom use, person disclosed to before study entry, membership in an HIV/AIDS association, location of HIV diagnosis, prior receipt of disclosure counseling, negative attitude toward one's HIV status, and feeling responsibility to disclose.

### Sample Size and Sampling Procedures

We estimated the sample size using a Fleiss formula for 2 proportions (Box).^41^

BOX **Equation for Estimating Sample Size**

$$N=\frac{[z_{\alpha }\sqrt{P(1-P)(1/q_{1}+1/q_{2})}+z_{\beta }\sqrt{P_{1}(1-P_{1})(1/q_{1})+P_{2}(1-P_{2})(1/q_{2}){]}^{2}}}{{\left(P_{1}-P_{2}\right)}^{2}}.$$

where p1 was assumed to be the proportion of control arm participants expected to disclose at the end of the study, p2 was the assumed proportion of intervention arm disclosure, q1 was the assumed proportion of non-disclosure in the control arm, and q2 was the assumed proportion of non-disclosure in the intervention arm. P = q1p1 + q2p2, and N was the total number of participants.

Considering the baseline disclosure of 54%,^42^ we assumed a disclosure increase of 46% by the intervention based on a previous study in which CHWs improved tuberculosis sputum positive case detection.^43^ Therefore, using the information above, we determined the expected proportion in the intervention group to be 0.78. We set power at 80% (Zβ=0.84), design effect of 2, and alpha at 0.05 (Zα=1.96). Thus, the total estimated sample size was approximately 236.

### Statistical Analysis

Data were collected using REDCap version 8.5.11 then transferred into an Excel spreadsheet and later to STATA version 15/MP for analysis.^44^ Univariately, data were summarized using descriptive texts and summarizing tables and graphs. Continuous variables like age were summarized as median with interquartile range while categorical variables were summarized as frequencies and proportions in tables. Cumulative disclosure prevalence was obtained by dividing the number of participants who disclosed at the end of the follow-up by the total number of participants who completed the study and multiplied by 100. Bivariate analysis was done using a clustered modified Poisson regression model with robust standard errors and this was fitted to get unadjusted relative ratios (RRs). All independent variables with a *P* value of <.2 at bivariate and those known to influence disclosure from the literature were entered into a multivariate clustered modified Poisson model to determine the independent factors associated with disclosure. Interaction and confounding were also assessed before fitting the final model. Confounding was determined if there was a >10% change in the adjusted and unadjusted RRs. Interaction was assessed only on significant variables at bivariate level.

### Ethical Approval

The protocol was reviewed and approved by the School of Medicine Institutional Review Board - Makerere University (REC REF 2019-100). Additional clearance was sought from the Uganda National Council for Science and Technology (HS443ES). The district health departments granted permission to undertake the study. All participants gave their informed written consent. Confidentiality and anonymity were strictly observed at all the research stages. All CHWs who were contacted and agreed to participate were trained on health ethics, confidentiality, and handling of adverse outcomes of disclosure. Additionally, we also obtained informed consent from the CHWs to participate in the study. Participant safety was ensured throughout the study. Participants who experienced adverse outcomes, such as quarreling and separation, were reconciled to the best of the team’s ability before their termination in the study. Partner HIV testing and referral to HIV care (for newly positive partners) were done upon their approval. All methods were done in accordance with relevant guidelines and regulations of good clinical practice and human subject protection.

## RESULTS

**FIGURE 2:**
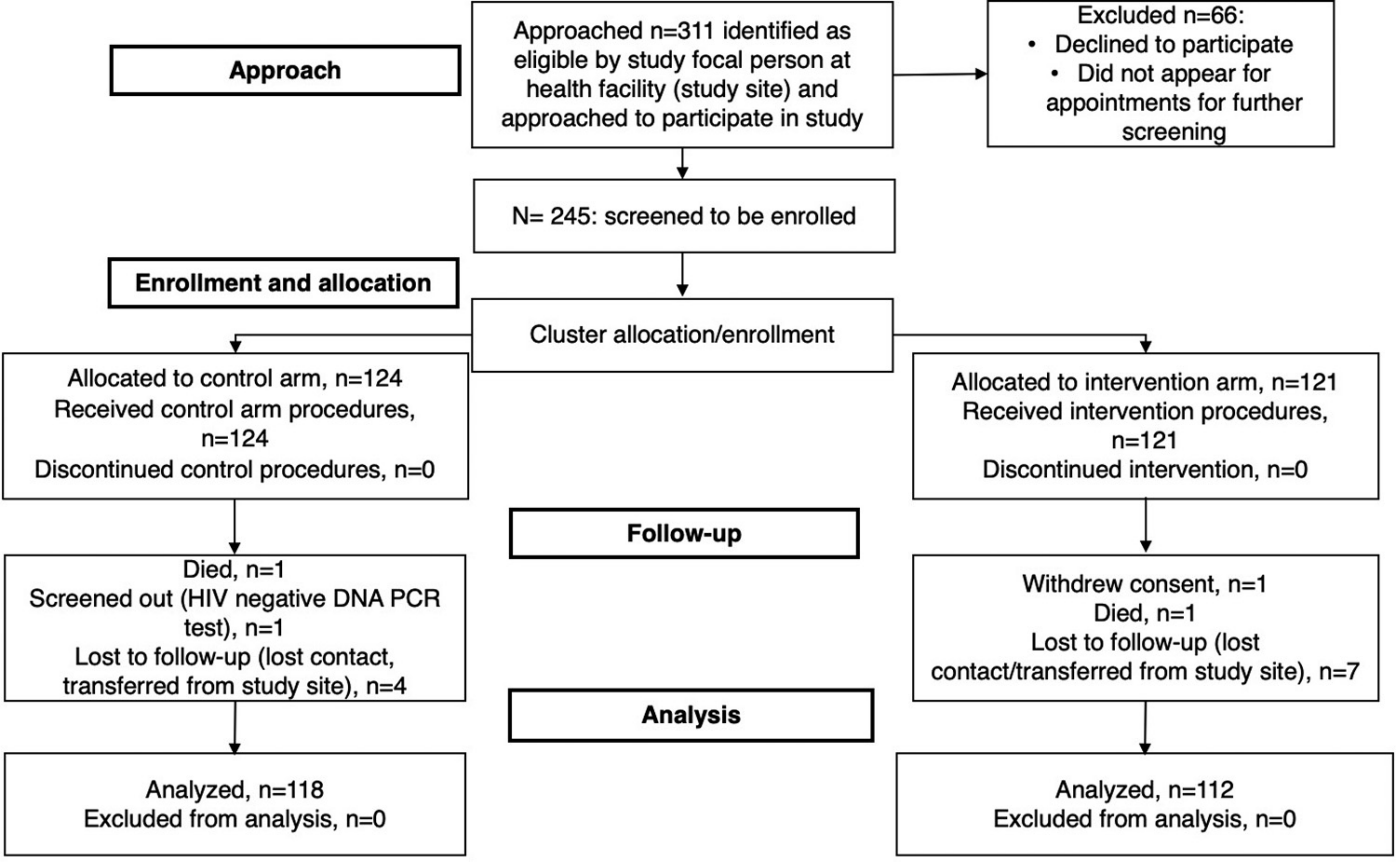
Number of Adults Living With HIV Evaluated at Each Study Stage in Rural Uganda Abbreviation: PCR, polymerase chain reaction.

A total of 245 participants were enrolled from 10 health facilities, with an average of 25 participants per facility. Two facilities enrolled participants in both study arms, and the rest enrolled in either the intervention or control arm. A total of 230 (93.9%) participants completed the study, and of these, 112 (48.7%) were in the intervention arm and 118 (51.3%) were in the control (Figure 2). The median age was 30 (interquartile range=25–37) years. The majority of those enrolled were women (76.5%) (Table 1).

**TABLE 1.** Demographic Characteristics of Adults Living With HIV in Rural Uganda

**Characteristic**	**Sample Size, No. (%) (N=230)**	**Intervention, No. (%) (n=112)**	**Control, No. (%) (n=118)**	***P* Value**
Age group, years				.102
18–35	163 (70.9)	85 (75.9)	78 (66.1)	
35–55	67 (29.1)	27 (24.1)	40 (33.9)	
Gender				.004
Female	176 (76.5)	95 (84.8)	81 (68.6)	
Male	54 (23.5)	17 (15.2)	37 (31.4)	
Education				.019
None	30 (13.0)	12 (10.7)	18 (15.3)	
Primary	122 (53.1)	52 (46.4)	70 (59.3)	
Secondary	67 (29.1)	39 (34.8)	28 (23.7)	
Tertiary	11 (4.8)	9 (8.0)	2 (1.7)	
Marital status				.008
Casual partner	60 (26.1)	22 (19.6)	38 (32.2)	
Cohabiting	139 (60.4)	68 (60.7)	71 (60.2)	
Married	21 (9.1)	22 (19.6)	9 (7.6)	
Nature of marriage				.019
Monogamous	13 (5.7)	8 (7.1)	5 (4.2)	
Polygamous	18 (7.8)	14 (12.5)	4 (3.4)	
Not mentioned	199 (86.5)	90 (80.4)	109 (92.4)	
Duration of relationship				.521
<6 months	14 (6.1)	5 (4.5)	9 (7.6)	
6 months–1 year	57 (24.8)	30 (26.8)	27 (22.9)	
>1 year	159 (69.1)	77 (68.8)	82 (69.5)	
Monthly income, UGX (US$)^a^				.989
<120000 (<31)	147 (63.9)	72 (64.3)	75 (63.6)	
120000–500000 (31–131)	77 (33.5)	37 (33.0)	40 (33.9)	
>500000 (>131)	6 (2.6)	3 (2.7)	3 (2.5)	

a Ugandan shilling (UGX)3819=US$1.

### Participant Clinical and Behavioral Characteristics

The majority of the participants (184, 80%) didn’t know their partners’ HIV status. Among sexual partners whose HIV status was known by participants, 38 (82.6%) were known as negative. A total of 144 participants (62.6%) had been on ART for at least a year. Overall, 191 participants (83.0%) had disclosed to either a friend or relative, and 39 (17.0%) had never disclosed to anyone. Only 13 participants (5.7%) were members of an HIV support group or association (e.g., as peer educators). Most participants (182, 79.1%) had received partner disclosure counseling from a health worker at their respective HIV care centers. The majority of the participants (120, 52.2%) reported having a negative attitude toward other people knowing their HIV-positive status (Table 2).

**TABLE 2.** Clinical and Behavioral Characteristics of Adults Living With HIV in Heterosexual Relationships in Rural Uganda

**Characteristic**	**Sample Size, No. (%) (N=230)**	**Intervention, No. (%)** **(n=112)**	**Control, No. (%)** **(n=118)**	***P* Value**
Partner HIV status				.049
Negative	38 (16.5)	12 (10.7)	26 (22.0)	
Positive	8 (3.5)	3 (2.7)	5 (4.2)	
Do not know	184 (80.0)	97 (86.6)	87 (73.7)	
Duration on ART				.001
<6 months	55 (23.9)	38 (33.9)	17 (14.4)	
6 months–1 year	31 (13.5)	10 (8.9)	21 (17.8)	
>1 year	144 (62.6)	64 (57.1)	80 (67.8)	
Condom use				.191
No	130 (56.5)	68 (60.7)	62 (52.5)	
Sometimes	83 (36.1)	39 (34.8)	44 (37.3)	
Always	17 (7.4)	5 (4.5)	12 (10.2)	
Person disclosed to before study entry				.050
None	39 (17.0)	24 (21.4)	15 (12.3)	
Friend	21 (9.1)	6 (5.4)	15 (12.7)	
Relative	170 (73.9)	82 (73.2)	88 (74.6)	
Place of HIV diagnosis				0.033
ANC clinic	59 (25.7)	32 (28.6)	27 (22.9)	
VCT clinic	149 (64.8)	75 (67.0)	74 (62.7)	
Other	22 (9.6)	5 (4.5)	17 (14.4)	
Membership in HIV/AIDS association				0.127
No	217 (94.3)	103 (92.0)	114 (96.6)	
Yes	13 (5.7)	9 (8.0)	4 (3.4)	
Prior receipt of disclosure counseling				.004
No	48 (20.9)	27 (24.1)	21 (17.8)	
Always	37 (16.1)	8 (7.1)	29 (24.6)	
Only at testing	75 (32.6)	42 (37.5)	33 (28.0)	
Sometimes	70 (30.4)	35 (31.3)	35 (29.7)	
Negative attitude toward other people getting to know one’s HIV status				.005
No	110 (47.8)	43 (38.4)	67 (56.8)	
Yes	120 (52.2)	69 (61.6)	51 (43.2)	
Feeling responsibility to disclose				.140
No	22 (9.6)	14 (12.5)	8 (6.8)	
Yes	208 (90.4)	98 (87.5)	110 (93.2)	
Ever had a chance(s) to disclose				
No	166 (72.2)	93 (83.0)	73 (61.9)	<.001
Yes	64 (27.8)	19 (17.0)	45 (38.0)	

Abbreviation: ANC, antenatal care; ART, antiretroviral therapy; VCT, voluntary counseling and testing.

Most participants had received partner disclosure counseling from a health worker at their respective HIV care centers.

### Factors Associated With HIV Disclosure

Participants in the intervention arm were 51% more likely to disclose compared to those in control (adjusted relative ratio [aRR]=1.51; *P*<.001). Male gender and membership in an HIV/AIDS support group increased disclosure by 24% (aRR=1.24; *P*=.004) and 18% (aRR=1.18; *P*=.044), respectively. However, prior receipt of disclosure counseling and having a negative attitude toward self-HIV-positive status reduced disclosure by 30% (aRR=0.69, *P*=.001) and 21% (aRR=0.79; *P*=.001), respectively (Table 3).

**TABLE 3.** Demographic, Clinical, and Behavioral Factors Associated With HIV Disclosure Among Adults Living With HIV in Heterosexual Relationships in Rural Uganda

**Characteristic**	**No. (%) (N=230)**	**Crude RR (95% CI)**	**Adjusted**^a^ **RR (95% CI)**	***P* Value**
Study arm				
Control	118 (51.3)	1.00	1.00	
Intervention	112 (48.7)	1.45 (1.24, 1.70)	1.51 (1.28, 1.77)	<.001
Gender				
Female	176 (76.5)	1.00	1.00	
Male	54 (23.5)	0.96 (0.81, 1.14)	1.24 (1.07, 1.44)	.004
Prior receipt of disclosure counseling				
No	48 (20.9)	1.00	1.00	
Always	37 (16.1)	0.82 (0.63, 1.07)	0.80 (0.61, 1.03)	.082
Only at testing	75 (32.6)	1.05 (0.91, 1.20)	1.06 (0.92, 1.22)	.438
Sometimes	70 (30.4)	0.68 (0.54, 0.86)	0.70 (0.56, 0.87)	.001
Negative attitude toward other people getting to know one’s HIV status				
No	110 (47.8)	1.00	1.00	
Yes	120 (52.2)	0.76 (0.66, 0.89)	0.79 (0.69, 0.91)	.001
Ever had chance(s) to disclose				
No	166 (72.2)	1.00	1.00	
Yes	64 (27.8)	1.16 (0.99, 1.37)	1.14 (0.97, 1.34)	.118
Membership in HIV/AIDS association				
No	217 (94.3)	1.00	1.00	
Yes	13 (5.7)	1.17 (0.99, 1.37)	1.18 (1.01, 1.39)	.044
Person disclosed to before study entry				
None	39 (17.0)	1.00	1.00	
Friend	21 (9.1)	0.71 (0.49, 1.03)	0.74 (0.50, 1.12)	.155
Relative	170 (73.9)	0.85 (0.74, 0.97)	0.91 (0.77, 1.07)	.249
Duration of relationship				
<6 months	14 (6.1)	1.00	1.00	
6 months–1 year	57 (24.8)	0.94 (0.76, 1.17)	1.04 (0.84, 1.27)	.741
>1 year	159 (69.1)	0.89 (0.77, 1.04)	0.97 (0.79, 1.18)	.755

Abbreviation: CI, confidence interval; RR, relative ratio.

a Adjusted for: age, gender, prior receipt of disclosure counseling, membership to HIV/AIDs association, fear of stigma, ever had chance(s) of disclosure, negative attitude toward other people getting to knowing one’s HIV status, and person disclosed to prior to the study entry.

### Disclosure Prevalence and Adverse Events Following Disclosure

**FIGURE 3:**
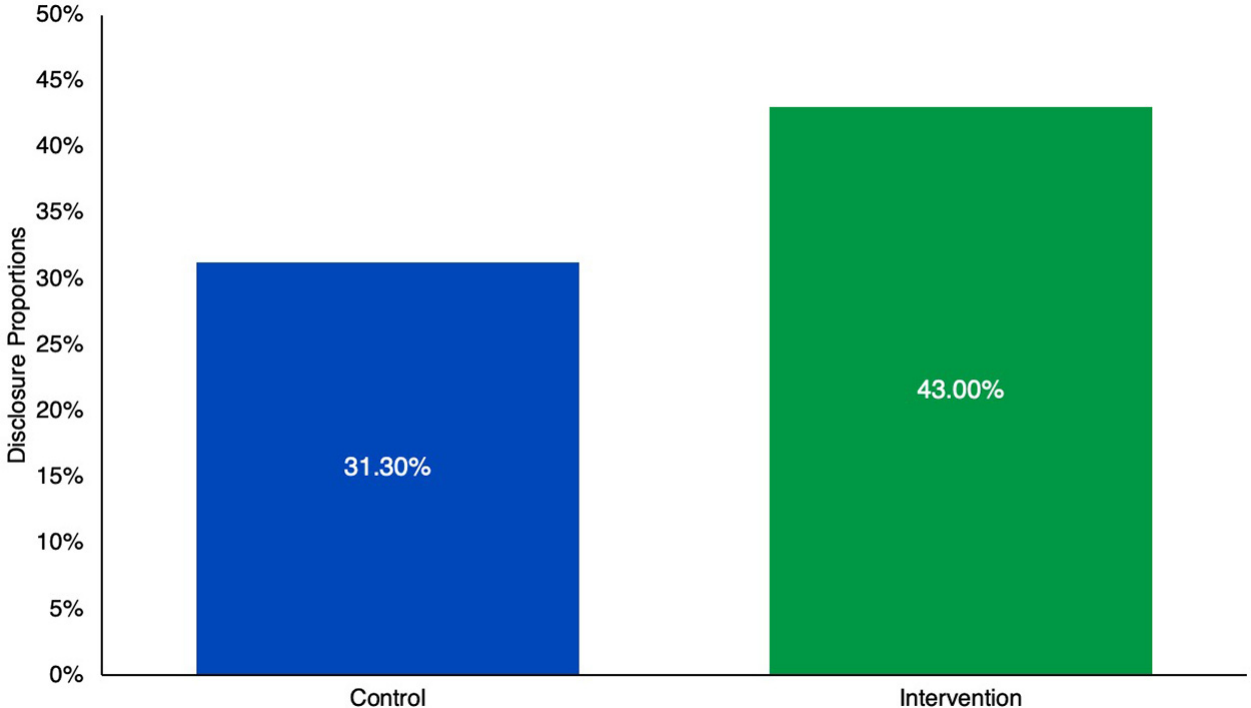
Disclosure Proportions Across Study Arms Among Adults Living With HIV in Rural Uganda^a^ ^a^*P*<.001.

A total of 171 participants disclosed their HIV status to their partners, giving a disclosure prevalence of 74.4% (95% confidence interval [CI]= 68.2, 79.9). Of 171 participants, 99 were from the intervention arm (43.0%; 95% CI=36.6, 49.7), and 72 from the control (31.3%; 95% CI=25.4, 37.7). The disclosure fraction attributed to the intervention was 0.31 (95% CI=0.19, 0.41; *P*<.001) (Figure 3).

Among the partners who were disclosed to, 104 (60.8%) came to the study site for HIV counseling and possible testing; of these, 55 (52.9%) tested negative, 23 (22.1%) confessed to be HIV positive and already in HIV care, and 26 (25%) newly tested positive (were linked to HIV care).

Of those that disclosed, 12 (12.1%) and 9 (12.5%) participants in the intervention and control arms, respectively, experienced adverse events. In the intervention group, adverse events included separation (5, 5.1%), quarrelling or abuse (4, 4.0%), and threatening (3, 3.0%). In the control group, adverse events included separation (1, 1.4%), quarrelling or abuse (4, 5.6%), and threatening (4, 5.6%) (Table 4).

**TABLE 4.** Adverse Events Following Disclosure Among Adults Living With HIV in Heterosexual Relationships in Rural Uganda

**Adverse Events**	**Intervention, No. (%)**	**Control, No. (%)**	***P* Value**
Total	12 (12.1)	9 (12.5)	.941
Separations	5 (5.1)	1 (1.4)	.199
Quarreling/abuse	4 (4.0)	4 (5.6)	.411
Threatening	3 (3.0)	4 (5.6)	.643

## DISCUSSION

Our CHW intervention increased HIV disclosure by 51% with an attributable disclosure fraction of 31.0%. This provides empirical evidence that CHWs could have a substantial role in supporting disclosure among adults living with HIV in heterosexual relationships with disclosure difficulties. Men were 24% more likely to disclose compared to women, and membership in HIV/AIDS associations increased disclosure by 18%. However, having a negative attitude toward self-HIV-positive status and prior receipt of disclosure counseling reduced disclosure by 21% and 30%, respectively.

Our intervention provides empirical evidence that CHWs could have a substantial role in supporting disclosure among adults living with HIV in heterosexual relationships with disclosure difficulties.

Through continuous community-based counseling, home visits, phone calls, and disclosure skills building, CHWs encouraged and supported adults living with HIV in the disclosure process and, hence, increased HIV disclosure. In Uganda, CHWs had specific roles and responsibilities, but supporting disclosure was not specifically documented.^30^ In addition to their already well-known support for HIV care services,^31^^,^^32^ CHWs have consistently offered support in various HIV care contexts. In Malawi, CHWs significantly improved the continuum of care in the prevention of mother-to-child transmission of HIV.^45^ In South Africa, they improved viral load suppression among PLHIV,^46^ and in sub-Saharan Africa at large, they improved quality of life for PLHIV.^32^ Although CHWs have not had a positive influence on HIV treatment outcomes in some settings,^47^^–^^49^ they have generally improved the delivery of health services, especially in rural settings.^34^^,^^50^^,^^51^ Therefore, our findings strengthen the importance of CHWs in improving health care services, particularly in HIV care.

HIV disclosure, particularly among sexual partners, is vital in HIV management because of its prevention and care benefits, such as improving ART adherence and retention in care and reducing HIV transmission.^8^^,^^9^^,^^12^^–^^14^ It also promotes social support and enhances trust and social acceptance.^10^^,^^11^ The community-based counseling approaches used by CHWs reduce HIV stigma and discrimination,^33^ which aids HIV disclosure. Therefore, having CHWs able and willing to support disclosure is a breakthrough in expanding community-based HIV care and management for PLHIV experiencing disclosure difficulties and hardships accessing health facilities or professional health care workers. Since CHWs already have documented responsibilities, adding a disclosure support task may require HIV care programs to train CHWs on HIV disclosure requirements. Although motivation and facilitation are among the key priorities in CHW strategy and operational guidelines, particularly in Uganda,^30^ CHWs always report hardships in executing their work and inadequate motivation and facilitation.^52^^–^^55^ Therefore, since disclosure is a process that requires time, patience, and commitment,^56^^,^^57^ there is a need to revive motivation and facilitation for CHWs,^58^ especially when adopting new tasks for them.

Men were more likely to disclose compared to women; this was similar to the findings in previous studies.^59^^–^^61^ Due to their financial independence and gender power to have higher self-efficacy and positive outcome expectancies compared to women,^62^^,^^63^ men are less likely to fear financial support implications, gender-based violence, or adverse events that may follow disclosure.^17^^,^^20^^,^^21^ However, many studies found that women were more likely to disclose than men,^64^^–^^66^ while others found no gender differences in HIV disclosure.^67^^,^^68^ These differences may be due to variations in the study setting, design, and population. However, for the current study, continuous community-based counseling and encouragement of men by CHWs may have influenced and motivated disclosure among men. Because men rarely receive adequate HIV disclosure counseling due to their low attendance at health facilities,^26^ the CHW mechanism may have reached them adequately in the community.

Participation in an HIV/AIDS association or group (e.g., as a peer educator) increased the chances of disclosure, a finding consistent with previous studies.^8^^,^^60^^,^^69^ Such groups create a sense of duty in an individual to inform others about one’s HIV status due to the occasional receipt of information regarding prevention of HIV transmission and adherence to HIV care services.^70^ Also, being leaders in some contexts, peer educators are meant to be exemplary to others; hence, they feel more responsibility to disclose compared to other PLHIV. In contrast, a multicenter study done in Burkina Faso, Kenya, Malawi, and Uganda showed that HIV support groups were negatively associated with HIV disclosure.^71^ This difference could have been due to the differences in study design, as that study was cross-sectional as opposed to the current longitudinal interventional study.

Having a negative attitude toward other people knowing one’s HIV status reduced the chances of disclosure. This is probably linked to HIV-related stigma, as the negative effect of stigma on disclosure has already been reported.^72^^–^^76^ In addition to stigma, fear of the negative consequences of disclosure in a sexual relationship might have been responsible for the negative attitude some participants exhibited toward other people knowing their HIV status, as also reported elsewhere.^77^^,^^78^ However, several cross-sectional studies reported perceived stigma as a non-significant factor for HIV status disclosure.^70^^,^^79^^,^^80^ The cross-sectional nature of these studies might be responsible for the contradiction with the current interventional study.

Having a negative attitude toward other people knowing one’s HIV status reduced the chances of disclosure.

Despite reports that receipt of disclosure counseling is associated with disclosure,^60^^,^^66^ participants who had received disclosure counseling before study entry were less likely to disclose. This could have been because these participants may have made disclosure attempts before the study and noticed the possible negative consequences, which they never wanted to elicit again.

It was remarkable that 22.1% of the partners who were disclosed to and discovered to be HIV positive were already in HIV care. This is similar to reports from previous studies that concordant positive sexual partners may not be aware of each other’s HIV status, and occasionally, they may suspect each other’s HIV positivity without open discussion about it.^37^^,^^81^

The overall prevalence of adverse outcomes following disclosure was 12.3%, and there was no significant difference between the study arms. However, this prevalence was remarkably lower than in previous studies.^59^^,^^82^^–^^85^ Specifically, the overall prevalence of separation was 3.5%, which was lower than the average of 8.3% reported in previous studies.^59^^,^^86^^–^^89^ In the current study, partner separations were more in the intervention than in the control arm (5.0% versus 1.4%), though not statistically significant. However, separations in the intervention arm were lower than the 9.0% and 7.7% reported in previous interventional studies.^82^^,^^86^ The findings in the current study reaffirm the fact that negative outcomes may occur following disclosure, but the risks are much smaller in the long term and are worth undertaking, as reported in previous studies.^10^^,^^11^^,^^90^

The findings in the current study reaffirm the fact that negative outcomes may occur following disclosure, but the risks are much smaller in the long term and are worth undertaking.

### Study Strengths and Limitations

To the best of our knowledge, this is the first study to scientifically evaluate the role of CHWs in supporting HIV disclosure among adults living with HIV in heterosexual relationships. We reduced the social desirability bias associated with HIV disclosure^15^ by confirming self-reported disclosures with CHWs in the intervention arm and encouraging those who disclosed to bring their partners to the study site or HIV care center for additional counseling and testing. Fortunately, 60.8% of the sexual partners who were disclosed to came to the study site (health facility) for further counseling and HIV testing.

The findings of this study should be interpreted with caution because this was a nonran-domized cluster study, which was prone to selection bias or confounding. However, confounding was assessed during statistical analysis and no confounder was identified; hence, there was minimal possibility of this occurrence. Because of the community nature of the intervention, the study was prone to cross-contamination from the possibility of sharing intervention information between the participants of the 2 study arms. However, we created a buffer zone between the clusters in the intervention arm and those in the control arm, which minimized the possibility of participants from different study arms meeting while in the community. To minimize contamination at the study sites during in-clinic study visits, participants in the intervention group at the sites that recruited in both arms were given different appointment dates for enrollment and follow-up, which reduced their chances of meeting at the study sites. Finally, there was a variation in participants’ baseline characteristics between study arms, which was probably due to the nonrandomized nature of the study clusters. This limitation was minimized by controlling the presumed independent variables in the modified cluster multivariate regression analysis.

## CONCLUSION

The CHW-led mechanism increased HIV disclosure among adults living with HIV in heterosexual relationships in rural Uganda. CHWs can play a fundamental role in supporting disclosure among adults living with HIV with disclosure difficulties in a rural community setting. Further studies assessing and evaluating the operational feasibility and sustainability of this approach may be required.

## Notes

**Peer Reviewed**

**First published online:** September 22, 2022.
